# Deep Aphantasia: a visual brain with minimal influence from priors or inhibitory feedback?

**DOI:** 10.3389/fpsyg.2024.1374349

**Published:** 2024-04-05

**Authors:** Loren N. Bouyer, Derek H. Arnold

**Affiliations:** School of Psychology, The University of Queensland, Brisbane, QLD, Australia

**Keywords:** Aphantasia, congenital aphantasia, visual imagery, Bayesian priors, perception

## Abstract

The authors are both self-described congenital aphantasics, who feel they have never been able to have volitional imagined visual experiences during their waking lives. In addition, Loren has atypical experiences of a number of visual phenomena that involve an extrapolation or integration of visual information across space. In this perspective, we describe Loren’s atypical experiences of a number of visual phenomena, and we suggest these ensue because her visual experiences are not strongly shaped by inhibitory feedback or by prior expectations. We describe Loren as having Deep Aphantasia, and Derek as shallow, as for both a paucity of feedback might prevent the generation of imagined visual experiences, but for Loren this additionally seems to disrupt activity at a sufficiently early locus to cause atypical experiences of actual visual inputs. Our purpose in describing these subjective experiences is to alert others to the possibility of there being sub-classes of congenital aphantasia, one of which—Deep Aphantasia, would be characterized by atypical experiences of actual visual inputs.

## Introduction

Most people can generate images that they experience in their mind’s eye. We authors cannot, and do not believe we have ever been able to. We can be described as Congenital Aphantasics (Zeman et al., 2015). We each obtain the minimum possible score on the VVIQ2 questionnaire (Marks, 1995), which measures the subjective intensity of imagined visual experiences. But there are large differences between our subjective imagined experiences. Derek can have detailed imagined audio experiences (hearing snippets of symphonies at will), and his dreamt audio and visual experiences seem fully realistic (like most Congenital Aphantasics, see Zeman et al., 2015; Dawes et al., 2020). Loren, however, reports that she cannot have imagined audio experiences, has no inner monolog, and she does not have audio or visual experiences while dreaming. Loren can experience imagined tastes and tactile sensations, Derek cannot.

From the outset, we want to be clear that this is a subjective perspective piece. Our descriptions should be regarded as anecdotal evidence. However, we think that personal reflective accounts, and case studies, continue to play an important role in psychological science—to inspire theory and to alert people to conceptual possibilities (for related examples and arguments, see Zeman et al., 2010; Cubelli and Della Sala, 2017; dos Santos et al., 2018). We hope to further that tradition here.

The diversity of Loren and Derek’s experiences adds to a growing body of evidence—that congenital aphantasia can manifest as diverse patterns of modality specific inability to have imagined experiences (e.g., Dawes et al., 2020; Takahashi et al., 2023). We believe these idiosyncratic patterns of modality specific inability will be an important target for investigations that seek to understand the neural pre-requisites for conscious awareness of imagined experiences. However, our primary concern here is to describe Loren’s atypical experiences of actual visual inputs.

Our immediate goal is to describe Loren’s experiences of some visual inputs, and to advance a hypothesis that we believe can account for these. Our broader goal is to alert others to the possibility of Deep Aphantasics, who in addition to being unable to have imagined sensory experiences will also have atypical experiences of actual sensory inputs. More specifically, we believe they will have atypical experiences of a number of visual phenomena that involve perception being shaped by expectations or by feedback.

## Aphantasia and perception

We note that there is existing evidence that Aphantasics can have atypical experiences of sensory inputs. For instance, Dance et al. (2021a) showed that Aphantasics are less likely to report experiencing hyper (e.g., bright lights causing a headache) or hypo (e.g., taking pleasure from listening to paper rustling) sensitivity to actual sensory inputs, and they suggested this might be due to Aphantasics being overall less responsive to inputs. This aligns with evidence that Aphantasics are less likely to report having experienced a flicker induced pseudo-hallucination than are members of the general population (Reeder, 2022). Loren’s atypical experiences of visual phenomena, however, cannot be accounted for by a simple propensity to be under responsive to inputs (Dance et al., 2021a). Rather, we will suggest the overall pattern of Loren’s visual experiences suggests that they are minimally impacted by expectations or by feedback.

## Loren’s perceptual experiences

**Figure 1:**
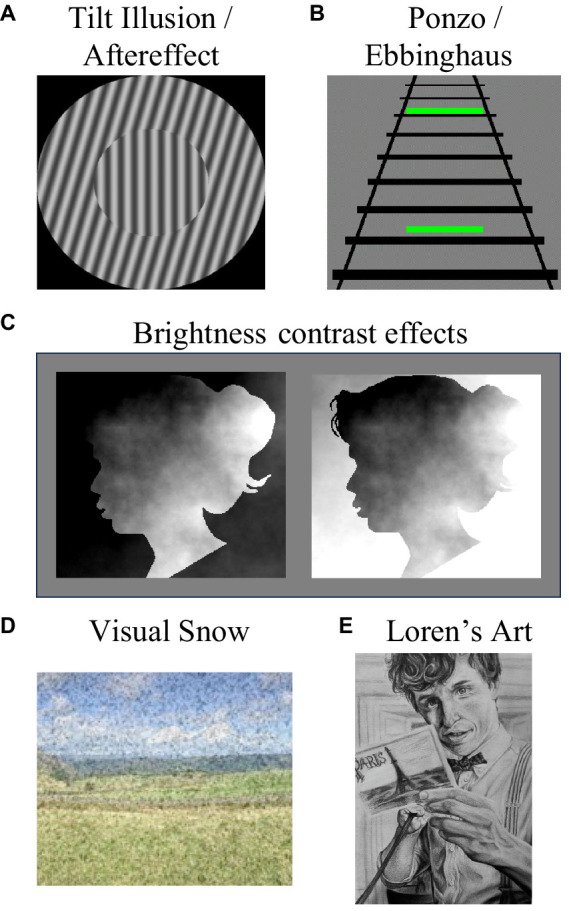
Some visual phenomena that Loren experiences. She experiences **(A)** tilt aftereffects and their spatial analog, the tilt illusion, **(B)** she experiences simultaneous size contrast illusions, such as the Ponzo and Ebbinghaus illusions, and **(C)** she experiences brightness contrast effects. Here the two facial images are physically identical, but on the left the face looks relatively bright as it features the brightest regions of the image, and on the right it looks dark as it features the darkest regions of the image. While Loren’s experiences of all these effects are stereotypical, she also experiences visual snow **(D)**. Loren is an accomplished artist, as demonstrated by her drawing **(E)**, but she cannot draw from memory.

Loren seems to have stereotypical experiences of visual phenomena that are likely to be driven by low-level processes. For instance, she experiences motion (Addams, 1834) and tilt (Gibson, 1937) aftereffects, which are both driven by visual adaptation (reduced neural responding after protracted exposure to an input, see Webster, 2015). Loren also experiences the tilt illusion (Gibson and Radner, 1937, see Figure 1A), which is driven by lateral inhibition between adjacent columns of orientation-tuned neurons (Blakemore et al., 1970). She also has stereotypical experiences of some simultaneous contrast illusions, such as the Poggendorff size/contrast illusion (Eagleman, 2001, see Figure 1B), and brightness contrast effects (e.g., Anderson and Winawer, 2005, see Figure 1C). Loren experiences variants of the peripheral drift illusion (e.g., Faubert and Herbert, 1999), which are driven by involuntary eye movements and brightness gradients combining to activate direction-selective cells (Bach and Atala-Gérard, 2020).

**Figure 2:**
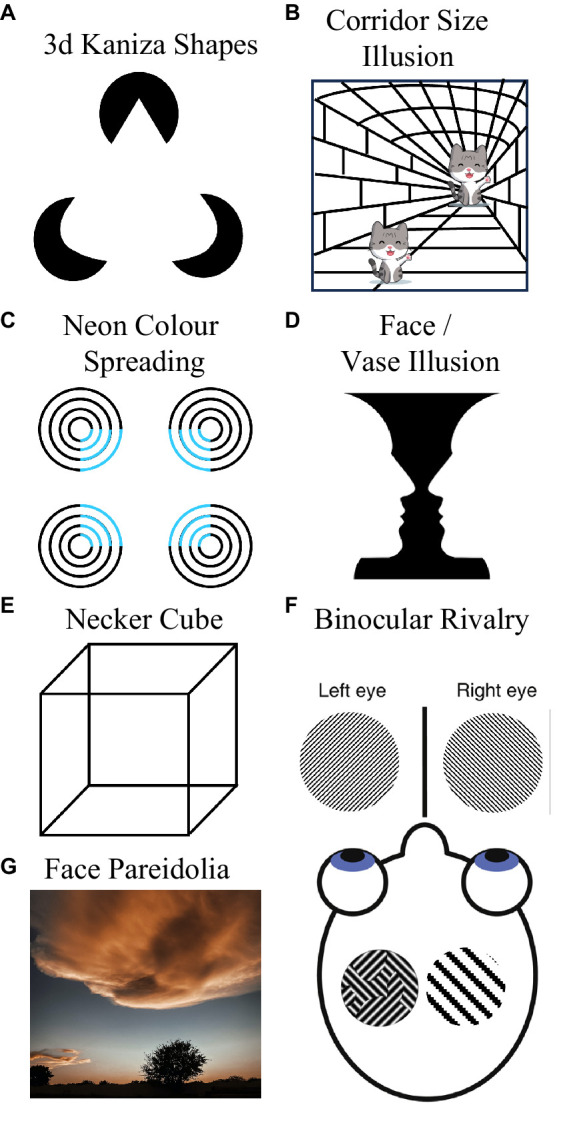
Some visual phenomena that Loren does not experience stereotypically. She cannot discern 3D Kaniza shapes **(A)**, she does not immediately experience the Corridor Size Illusion **(B)**, she does not experience neon color spreading **(C)**, she cannot see the vase in Rubin face/vase illusion **(D)**, she cannot see a 3D cube in the line drawings of Neckers cube **(E)**, she does not experience periods of dominance during binocular rivalry **(F)**, and she does not experience face Pareidolia **(G)**.

Loren does not, however, have typical subjective experiences of a number of visual phenomena that involve extrapolation or integration of visual information across space. Loren cannot discern 3D kaniza shapes (Van Tonder and Ohtani, 2008). To Loren, the 3D cone depicted in Figure 2A looks like a weirdly shaped triangle. Loren can experience the corridor size illusion (Gregory, 1966, see Figure 2B), but only after a delay during which the depicted figures seem matched in size (people typically instantly experience these as very different in size). Loren does not seem to experience neon color spreading—she cannot see an illusory floating blue square in Figure 2C. Loren does not experience variants of long-range apparent motion either. When offset disks are flashed on and off in counterphase (see Anstis et al., 1985), she tends to experience localized flashes—she does not experience a sense of movement in-between the flash positions—as is typical.

Loren does not have typical experiences of bi-stable visual phenomena. These typically promote intermittent switching between two mutually exclusive perceptual experiences. For instance, Rubin’s (1915) vase (see Figure 2D) is typically experienced as either a pair of faces, or as a vase. However, Loren typically only sees the faces (and not the vase). Nor can Loren discern Necker’s (1832) cube. To her this just resembles a jumble of 2D shapes (see Figure 2E), so she does not experience switching between seeing one of the cube’s faces as being positioned at the front or at the rear of a 3D object. As Loren does not experience long-range apparent motion, she does not experience bi-stable long-range apparent motion (with movement seeming to alternate between moving in orthogonal directions, see Knapen et al., 2011).

When looking at kinetic silhouette illusions (see Troje and McAdam, 2010), that cause most people to experience intermittent reversals in the perceived direction of rotation, Loren experiences an unchanging (clockwise) direction. Loren does not have typical experiences of binocular rivalry. Inputs that elicit binocular rivalry in most people, characterized by alternating periods where only one of two rivalrous monocular images can be seen at a time, are only ever experienced by Loren as a fusion of the two images (transparency) or as a patchwork of both images (piecemeal rivalry, see left side image within the head of Figure 2F; Blake and Logothetis, 2002). Loren does not experience periods of dominance (where only one of the two rivalrous images can be seen), even after protracted viewing.

Loren does not experience face pareidolia (Rekow et al., 2022). To her non-face objects look like non-face objects, even if they feature two horizontally separated dark regions and an underlying feature that most people could regard as a nose or mouth (see Figure 2G). Loren feels like she is probably boring to go cloud watching with, as to her clouds are… just clouds. She does not see whimsical impressions of form in them. Finally, Loren experiences broadband visual snow—constant dynamic black and white dots across her visual field (see Figure 1D), and she thinks these might be associated with slight headaches.

Loren is an accomplished artist. She can draw realistic portraits, but only if she can see the input (see Figure 1E). Like other Aphantasics (Bainbridge et al., 2021), Loren cannot draw from memory. Neither Loren nor Derek have a history of neurological trauma, or diagnosis of a cognitive disorder. Loren’s structural brain scan did not reveal any overt abnormalities.

## Aphantasia and autism spectrum disorder

There are parallels between Loren’s visual experiences and those reported by people with Autism Spectrum Disorder (ASD, see Pellicano and Burr, 2012; Di Criscio and Troiani, 2017). However, even in that context some of Loren’s experiences stand out (we have not found any mention in that literature of an inability to *see* either the Necker cube or neon-color spreading) and others are more pronounced (e.g., her inability to experience bistable visual phenomena at all, as opposed to the slowed alternations that have been reported in ASD; see Di Criscio and Troiani, 2017).

We do not believe Loren meets criteria for a clinical diagnosis of ASD. Loren is socially communicative. She does not experience an impairment in reciprocal social communication and interaction that limits or impairs her everyday functioning—and these are essential features of ASD according to the Diagnostic and Statistical Manual of Mental Disorders (5th Edn., DSM-5-TR, American Psychiatric Association, 2013). With that said, we are mindful of criticisms regarding the identification of ASD in women and individuals with high IQs (Lai and Baron-Cohen, 2015; Rynkiewicz et al., 2016), and we are highly interested in evidence that Aphantasics express more autistic traits than do people in the general population (Dance et al., 2021b).

To some extent, links between Aphantasia and autistic traits are unsurprising, in that the most popular metric of Autistic traits in the general population, the AQ (Baron-Cohen et al., 2001), includes a subscale that measures the imagination (although one study that linked Aphantasia to AQ scores excluded a subscale question that is directly related to imagery, see Dance et al., 2021b). While Derek scores in the bottom 25% on the Autism Spectrum Quotient (AQ, Baron-Cohen et al., 2001), Loren’s score (37) is indicative of significant Autistic traits. However, we note that more recently developed metrics of Autistic traits (e.g., Ritvo et al., 2011; English et al., 2021) do not encompass an imagery subscale, and that more emphasis is now being placed on atypical sensory experiences. We believe further research on the interrelationship of ASD and Aphantasia is needed, and it should encompass multiple metrics of autistic traits. Indeed, we ourselves are engaged in that pursuit.

## Our hypothesis—Deep Aphantasia

The pattern of Loren’s visual experiences suggests to us that her visual brain activity is weakly shaped by prior expectations or by inhibitory feedback from frontal or semantic brain regions. We suspect the former because Loren does not have typical experiences of a number of phenomena that would seem to involve visual perception being shaped by prior expectations and experience (e.g., long-range apparent motion, 3D kaniza shapes, neon color spreading, and face pareidolia). This hypothesis is related to the idea that abnormal visual experiences in ASD are minimally impacted by prior expectations (see Pellicano and Burr, 2012). We suspect that attention fails to entrain reiterative inhibitory feedback to and within Loren’s primary visual cortex, as visual snow has been linked to hyperexcitable primary visual cortices due to an absence of top-down noise canceling inhibition (Hepschke et al., 2022). Also, top-down attention hypothetically drives the inhibitory interactions that cause alternations during bistable visual perception (e.g., Zhang et al., 2011), and Loren does not seem to experience bi-stable visual phenomena.

It is thought that Aphantasia results when feedback originating from frontal brain regions fails to excite activity in earlier brain structures that generate imagined sensory experiences (Pearson, 2019; Bartolomeo et al., 2020). We tentatively describe Loren as having Deep Aphantasia, and Derek as shallow, because for Loren a paucity of feedback might disrupt activity at a sufficiently early locus to perturb her experiences of *actual* visual inputs. For Derek, only imagined sensory experiences seem to be impacted. He has typical experiences of all the visual phenomena depicted in Figure 2.

We suspect Loren’s lack of imagined sensory experiences, and her atypical experiences of actual visual inputs, are inter-related because they could both reflect on a paucity of feedback. A paucity of feedback has been linked to visual snow (Hepschke et al., 2022), to mitigated inhibitory interactions during bi-stable visual perception (Zhang et al., 2011), and it has been implicated as a cause of Aphantasia (Pearson, 2019; Bartolomeo et al., 2020). Of course, we could be wrong. Loren’s cluster of atypical imagined and actual perceptual experiences could be coincidental and unrelated to a common cause. In that case, we would not expect numbers of people to self-identify as having a similar clustering of experiences.

## Advice for newly identified aphantasics

There has been some concern that identifying people as aphantasic could be stigmatizing (Blomkvist and Marks, 2023). Clearly, we authors are not personally concerned at this. While Loren is enthusiastic about the possibility of learning to have imagined visual experiences—to augment her artistic pursuits, Derek has existed with reasonable satisfaction for 50 years without visual imagery. He is consequently content with his existing cognitive profile. We encourage people who are newly identified as an Aphant to be similarly tranquil about their neurodiverse status.

There is evidence for cognitive differences between Aphants and neurotypical people. For instance, Aphants have less visually detailed long-term memories (e.g., Bainbridge et al., 2021; Dawes et al., 2022), and can be slower to make spatial judgments about imagined scenes (e.g., Liu and Bartolomeo, 2023). But while Aphants might be worse at some tasks, there is evidence we suffer less distress from reading fear evoking passages (Wicken et al., 2021)—which might relate to a greater resilience against adverse outcomes from intrusive thoughts (Brewin et al., 2010). So, you might be unable to conjure visual hallucinations at will, but that might not be such a bad thing.

## Then why are we interested in Aphantasia?

In addition to Loren’s desire to discover if visual imagery can be learned, we are greatly interested in Aphantasia as it seems to present an exciting research opportunity. By comparing the morphology and activity of human brains that can, and cannot, conjure imagined sensory experiences at will, we hope to discover pre-requisites for conscious awareness of imagined sensory experiences. By developing better means of identifying aphantasics, we might also gain insight into people’s capacity to benefit from psychological interventions that involve imagery (e.g., Jones and Stuth, 1997; Odou and Vella-Brodrick, 2013). Finally, while we would argue that neither Loren nor Derek have an impairment of reciprocal social communication that limits or impairs our everyday functioning, we acknowledge that we might have genetic similarities with people who do—and we regard that as an additional motivation to study Aphantasia.

## The VVIQ2

Given the prominence of the VVIQ2 (Marks, 1995) in contemporary research, we believe a brief comment on it is warranted. We join others (e.g., Blomkvist and Marks, 2023; Schwarzkopf, 2024) in arguing that the field needs a better means of identifying who is, and is not, aphantasic. The VVIQ2 is a subjective questionnaire, asking people to rate the vividness of their imagined visual experiences. Highlighting our concerns about the ambiguity of this instrument to aphantasics, when Loren first completed the VVIQ2 (Marks, 1995) her responses were stereotypical, as she was not then aware that other people could have imagined visual experiences. She misconstrued questions as relating to effort expended and success in remembering facts about visual experiences. Researchers have reported on other potentially diagnostic tasks (e.g., Chang and Pearson, 2017; Kay et al., 2022), but these have only ever been validated by correlation with the VVIQ2 (Marks, 1995). So, we have not really escaped reliance on identifying aphantasics via subjective report. We highlight this as a persistent issue that needs to be addressed.

## Conclusion

We have sought to document Loren’s subjective experiences, as we are confident that if these are made known, other people will self-identify as having similar experiences. We are hopeful that this will create opportunities to develop *objective* tests to identify and separate Shallow from Deep Aphantasics.

## Data availability statement

Information for existing publicly accessible datasets is contained within the article.

## Ethics statement

Written informed consent was obtained from the individual(s) for the publication of any potentially identifiable images or data included in this article.

## Author contributions

LB: Writing – review & editing. DA: Writing – original draft.
